# Biochemistry of Wine and Beer

**DOI:** 10.3390/biom11010059

**Published:** 2021-01-05

**Authors:** Encarna Gómez-Plaza, Rocío Gil-Muñoz

**Affiliations:** 1 Department of Food Science and Technology, Faculty of Veterinary Science, University of Murcia, Campus de Espinardo, 30100 Murcia, Spain; 2 Instituto Murciano de Investigación y Desarrollo Agrario y Alimentario (IMIDA), Ctra. La Alberca s/n, 30150 Murcia, Spain

Today, the production of wine and beer is a worldwide industry worth millions of euros annually, with breweries and wineries throughout the globe. The knowledge of the biochemical processes that occur prior and during beer and wine elaboration is large, but new technologies and analytical methodologies may provide completely new information. For this reason, this Special Issue tried to collect and present the various and latest findings of this field.

Many scientists have contributed to this Special Issue, which includes 15 papers, among them 11 original articles as well as four review articles that give readers of *Biomolecules* updated and new perspectives about the biochemistry of wine and beer.

Regarding beer research, five research articles have been published, covering methodologies for improving barley and malt quality, as reported by Pater et al. [1] who studied the quality of water treated with low-temperature, low-pressure glow plasma in order to obtain high-quality brewer’s malt, the use of not so-common yeast as the study of Zdaniewicz et al. [2] who used a non-*Saccharomyces* yeast, such as *Lachancea thermotolerans*, in the process of fermenting beer wort, analysing a broad number of quality parameters and concluding that the studied strain demonstrated a high capacity for rapid initiation of wort fermentation, and a tolerance to hop-derived compounds.

Also, in this special issue, three papers covering, from different point of views, the positive effect that beer may have on human health, can be found. In this way, Martinez-Gomez et al. [3] detailed a study of antioxidant compounds present in beer, focusing on the two main groups: phenols (including polyphenolic forms) and melanoidins, formed specifically during brewing as Maillard products. New trends to increase beer antioxidant activity were also discussed. Dulinsky et al. [4] discussed the potential for application of phytases in beer production, especially for the obtention of a buckwheat wort with high bioactive cyclitols. Finally, the possible effects of hop-derived acids on human cognitive functions were reviewed by Ayabe et al. [5]. They discussed the latest reports on the effects and underlying mechanisms of hop-derived bitter acids found in beer indicating that some beer compounds such as iso-α-acids and matured hop bitter acids enhance hippocampus-dependent memory and prefrontal cortex-associated cognitive and suggesting that these bitter acids enhance cognitive function via vagus nerve stimulation.

Regarding wine research, different strategies are still being tested in an attempt to optimize the use of available resources in an environment-safe scenario and to improve the polyphenolic quality of cultivated winegrapes, in order to obtain wines with better organoleptic and functional characteristics. Campayo et al. [6] reported the use of ozone as an alternative phytosanitary treatment to control grapevine diseases in order to reduce the use of conventional pesticides. This study showed that the application of ozonated water to Bobal grapevines not only affected the varietal aroma and polyphenolic content of wines. Elicitors can also be used in grapevines to increase the phenolic content in grapes and wines, so Paladines-Quezada et al. [7] combined the application of methyl jasmonate, benzothiadiazole, and methyl jasmonate + benzothiadiazole to Monastrell leaves and this in-field treatment was combined with pre-fermentative cold maceration at the beginning of winemaking. The results showed how the differences between the climatological conditions between the two years studied influenced in the results obtained due to the elicitors applications. Another solution for the adaptation of viticulture to climate change and to decrease grapevine susceptibility to cryptogamic diseases (such as mildew and downey) is to create new hybrid grape varieties combining durable resistance to downy and powdery mildews with a berry quality suitable for the production of high quality wines. González-Centeno et al. [8] showed a characterization of nine monovarietal wines from new red grape varieties, so-called Bouquet varieties, resistant to cryptogamic diseases (downy and powdery mildews) and these wines were evaluated in terms of their total phenolic, anthocyanin and proanthocyanidin contents, anthocyanin profile, volatile composition, and sensory attributes. These wines presented enough potential to become quality wines,

Regarding technological point of view, during the winemaking different tools can be used in order to obtain a high-quality wine. Traditionally, *Saccharomyces cerevisiae* has been the only yeast used during winemaking, but lately, non-*Saccharomyces* yeasts are also being used. Bindon et al. [9] reported a study on the role of *Saccharomyces cerevisiae* yeast strains (and their hybrids) on wine sensory properties. Specifically, the experiment was carried out with 10 commercially available yeast strains which were selected on the basis of their wide spread usage and/or novel properties. The results confirm previous reports demonstrating that the choice of yeast strain represents an opportunity to shape wine style outcomes. On the other hand, Morata et al. [10] have contributed with a very interesting review about non-*Saccharomyces* yeast strains of enological interest, especially for the recovering of the wine freshness lost due to the consequences of climate change. These authors also covered the influence of non-*Saccharomyces* yeasts on wine aroma, on the pH and acidity of the wines, on the formation of stable pyranoanthocyanin and polymeric pigments, which influence the wine color, and on the production of off-flavour; moreover, they reviewed those commercially available non-saccharomyces yeasts.

With respect to the wine, different aspects have been treated in this special issue. Osete-Alcaraz et al. [11] studied the interactions between cell wall components and wine tannins and evaluated the possible effect of polysaccharides in avoiding these interactions. The results showed that the addition of the commercial polysaccharides at the beginning of winemaking could lead to wines with higher phenolic content and improved chromatic characteristics, which remained stable after six months in the bottle, as seen from the highest scores reached in the sensory analysis. Aroma wine is another important aspect related to their organoleptic characteristics. Ferreira et al. [12] illustrated in a review the knowledge related to the aroma of grapes and to the aroma of wine with specific origin in molecules formed in grapes. Both grape aroma and grape-derived wine aroma are formed by a relatively large group of odorants belonging to different chemical and biochemical families. Only in the specific cases of aromatic grapes are there clear impact compounds or families of compounds defining the aroma profile. Additionally, grape-derived wine aroma molecules accumulate in quite different time periods of winemaking; some of them are mostly released during fermentation, while some others accumulate only after long periods of aging. Gaspar et al. [13] also studied the odor relevance of sotolon aroma in Madeira wine blends. The results showed that sotolon has an important contribution to the aroma of commercially available Madeira wine blends, particularly in those with long aging periods. Aging in barrels is other important step in quality wines, Rasines-Perea et al. [14] studied the polyphenolic compounds from oak wood, and particularly C-glucosidic ellagitannins, in 185 commercial samples from different areas (Bordeaux and Rioja), different barrel oak wood (French oak barrels and American oak barrels) and different ageing periods. The results showed differences between the two geographical zones in terms of compound concentrations.

Finally, the reultilization of by-products of the wine industry is studied by Moldovan et al. [15], who aimed to optimize the phenolic compounds extraction parameters from two grapes by-products (pomace and canes) as well as to evaluate the content of bioactive compounds and their antioxidant capacity. It was found that the conditions for the maximum polyphenols extraction from grape pomaces and canes would be 50% ethanol, 80 °C for 30 min, for all samples rich in phenolic antioxidant active principles.

As the Guest Editors for this Special Issue, we really would like to thank all the authors and co-authors for their contributions, all reviewers for their effort in revising and improving the manuscripts, as well as the editorial office of *Biomolecules* for their great help in organizing this Special Issue which we really hope will provide readers with an attractive opportunity to obtain new information concerning the different facets of wine and beer biochemistry.
